# Molecular characterization and phylogenetic analysis of dengue virus type 1 in Guangdong in 2014

**DOI:** 10.1186/s40064-016-3604-4

**Published:** 2016-11-09

**Authors:** Pei Wang, Huiling Wang, Jianhai Yu, Qian Xie, Zhiwei Yao, Zhiran Qin, Weizhi Lu, Jia Li, Minyi Zhang, Guangjin Cao, Zhicheng Zhong, Tianwen He, Danjuan Ma, Bao Zhang, Wei Zhao

**Affiliations:** 1 Guangdong Provincial Key Laboratory of Tropical Disease Research, School of Public Health, Southern Medical University, Guangzhou, 510515 China; 2 Department of Laboratory Medicine, The Second People’s Hospital of Guangdong Province, No. 466 Xingangdong Road, Guangzhou, 510317 China; 3 The Third Department of Infectious Disease, Guangzhou Eighth People’s Hospital, Guangzhou Medical University, Guangzhou, 510060 China; 4 Guang Dong Medical University Teaching Hospital, Guang Dong No. 2 People′s Hospital, Guangzhou, 510317 China; 5 Medical Genetical Center, Guangdong Women and Children Hospital, Guangzhou, 511442 China; 6 Department of Clinical Laboratory, Guangdong Provincial Maternity and Child Care, Guangzhou, 510010 China

**Keywords:** Dengue virus type 1, E gene, Phylogenetic analysis, Virus isolation

## Abstract

**Background:**

Dengue is one of the most important emerging diseases of humans, with no preventive vaccines or antiviral cures available currently. In 2014, the Southeast Asian region experienced an unprecedented outbreak of dengue, especially in Guangdong, China.

**Results:**

The nucleotide sequences of the E gene from 23 patients sera of dengue virus type 1 (DENV-1) from Guangzhou, China, were determined. One isolate that was recovered from a patient with serious liver damage was designated GZ02. The whole genome sequence of GZ02 was amplified, and confocal microscopy and plaque reduction neutralization test were performed to investigate the replication kinetics in liver L02 cells. In the study, assembly and genetic comparisons showed 11 of those E gene nucleotide sequences were absolutely accordant, and the nucleic acid sequence divergence among the other strains had no marked difference.

**Conclusions:**

Phylogenetic analysis based on the E gene indicated that the 23 new strains were closely related to strains from Malaysia or Singapore. Two different genotypes (genotype I and III) of DENV-1 were co-circulating in Guangdong, Malaysia, and Singapore from 2013 to 2014. However, no recombination event was found after 2005 between DENV strains from Guangdong and Malaysia or Singapore. GZ02 had a significant replicative advantage over DG14 and the DV1 standard strain. Importation of DENV-1 from Southeast Asian countries may have been an important contributing factor to the 2014 outbreak in Guangdong.

## Background

Dengue virus (DENV) is a member of the genus Flavivurs, family Flaviviridae. Dengue is a mosquito-borne viral disease that has spread throughout the world in recent years (Conceição et al. 2010). The incidence of dengue has increased remarkably in recent decades (Singh 2014). Currently, more than 40% of the world’s population is at risk for dengue virus (DENV) infection, and there is no preventive vaccine or effective antiviral treatment (WHO 2009). Dengue is of particular importance in Southeast Asia, where it has spread to 10 of 11 countries, and where 52% of the region’s population is at risk of DENV infection (Kyle and Harris 2008). With increasing travel, DENV continues its rapid spread worldwide (Wilder Smith and Schwartz 2005; Shu et al. 2009; Wilder-Smith 2012; Jiang et al. 2013; Zhao et al. 2014).

DENV is a single-stranded, positive-sense RNA virus with a genome of nearly 11 kb with a single open reading frame that encodes three structural proteins (C, capsid; prM/M, precursor of membrane; E, envelope), and seven non-structural proteins (NS1, NS2a, NS2b, NS3, NS4a, NS4b, NS5) (Conceição et al. 2010). DENV is divided into four antigenically related serotypes (DENV-1, DENV-2, DENV-3, and DENV-4) that show nucleotide sequence differences of almost 35% (Lee et al. 2012). To analyse the molecular evolution of the virus, a phylogenetic tree can be constructed using specific regions of the DENV genome, such as the full-length sequence, E, or E/NS1 (Huang et al. 2012; Guzman and Isturiz 2010; Holmes and Twiddy 2003). Studies on imported dengue cases can provide useful information about the geographic distribution and global movement of DENV strains. In 2014, a large outbreak of dengue, the cause of which remains unknown, occurred in Guangdong, China. Whether dengue fever is endemic in Guangdong is controversial. Some epidemiological investigations have revealed that Southeast Asia is an important source for DENV in Guangdong (Zaki et al. 2008). The close business relationships and air travel between Southeast Asian countries are thought to be responsible for the continuous importation of multiple DENV strains and the yearly outbreaks. Both strains from local cases and imported strains of DENV were isolated in 2014 (WHO Media Centre 2016). Some studies have suggested that the diversity of serotype and genotype within one geographic region may lead to the outbreaks of dengue (Sun and Meng 2013; Roehrig et al. 2008). We hypothesized that local circulation of the virus in Guangdong or the introduction of the virus from Southeast Asian countries may have led to the outbreak in Guangdong in 2014.

In the study, we analysed the origin of the 23 new dengue strains and reported the complete genome sequence of a DENV-1 strain (GZ02) isolated from the 2014 outbreak. This study aimed to investigate the origin of the Guangzhou outbreak strains, and to characterize these strains.

## Results

### Sequence analysis

In 2014, Guangzhou experienced a large outbreak of dengue fever. A total of 23 sequences of E gene from acute-phase patient sera were analysed. The E gene nucleotide sequences from 23 strains were determined and deposited in GenBank (Accession Nos. KR028435, KT382295–KT382305, KT428605–KT428615) (Table 1). Through comparative analysis, the sequences of 11 strains (GZ114, GZ17, GZ18, GZ19, GZ32, GZ43, GZ46, GZ61, GZ71, GZ75, and GZ79) were found to be identical. The nucleic acid sequence divergence among the GZ95, GZ87, GZ28, GZ03, GZ82, and GZ78 strains and those 11 strains above ranged from one to three bases. The E gene sequence of GZ93, GZ55, and GZ54 were identical, and GZ92, GZ76, and GZ14 showed only two nucleotide differences between them.

**Table 1:** List of the 23 nucleotide sequences of E gene during the 2014 outbreak in Guangzhou were used for phylogenetic study

Strains	GenBank accession	Genotypes
GZ114	KT382303	III
GZ17	KT428611	III
GZ18	KT382305	III
GZ19	KT382302	III
GZ32	KT382300	III
GZ43	KT428605	III
GZ46	KT382299	III
GZ61	KT428606	III
GZ71	KT428612	III
GZ75	KT428607	III
GZ79	KT428615	III
GZ95	KT428614	III
GZ87	KT428613	III
GZ28	KT382301	III
GZ03	KT428610	III
GZ62	KT382296	III
GZ78	KT428608	III
GZ93	KT382295	I
GZ55	KT382297	I
GZ54	KT382298	I
GZ92	KT428609	I
GZ76	KT428609	I
GZ02	KR028435	I

In the 2014 outbreak, a 31-year-old female was hospitalized in Guangzhou because of fever, skin rash, dizziness, and fatigue. PCR and IgM testing for DENV were positive. Liver function of this patient was abnormal which Alanine aminotransferase (ALT) and aspartate aminotransferase (AST) were significantly elevated, with no underlying disease like viral hepatitis. For further etiological study of this isolate, 12 pairs of primers were designed to amplify 12 overlapping fragments across the full length of the virus genome. The accession number of GZ02 in GenBank is KR028435; the full-length sequence was 10,671 nucleotides.

### Phylogenetic analysis

**Fig. 1:**
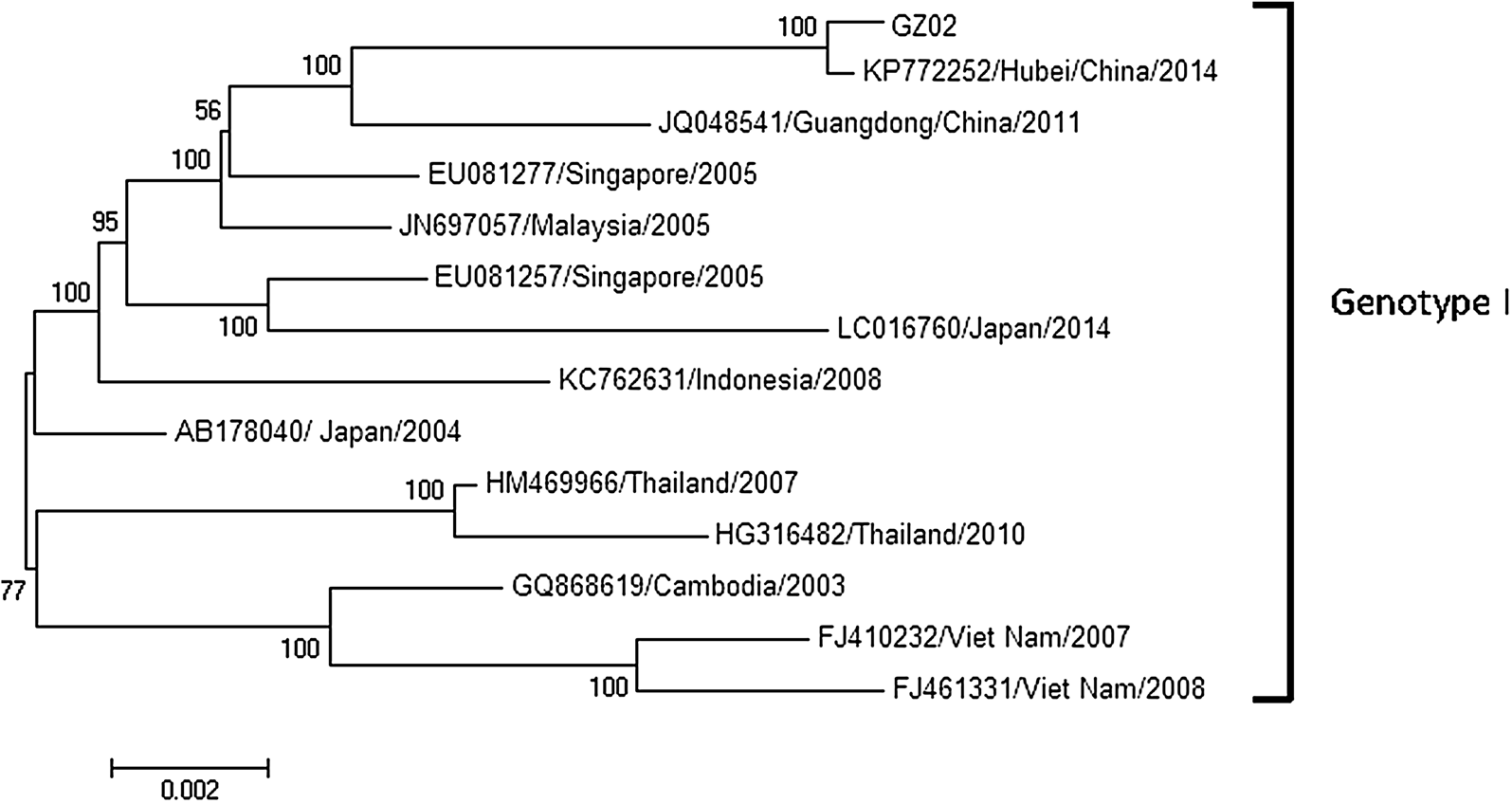
Phylogenetic tree constructed from full-length DENV sequences from different countries. A phylogenetic tree with DENV strains from different countries was generated by the neighbour-joining method, based on the complete nucleotide sequence of the open reading frame

Figure 1 is the full length analysis of isolate GZ02. This sequence and sequences from a representative strain from each country that might have introduced DENV into China were chosen to construct a phylogenetic tree (Sang et al. 2015). Most sequences included in the phylogenetic analysis came from Southeast Asian countries between 2003 and 2014 and have relatively high genetic nucleotide and amino acid similarity with GZ02 (Table 2). The phylogenetic tree of the full-length genome sequences showed that GZ02 is closely related to the DENV that circulates in Singapore, Malaysia, and Guangdong, China. The alignments of GZ02 and representative strain from each of these countries showed four nonsynonymous amino acid mutations in E and NS3 (Table 3). However, to date, there has been no report suggesting that these four sites affect DENV virulence or infectivity.

**Table 2:** Nucleotide (NT) and amino acid (AA) identities between GZ02 and DENV isolates from different countries

Strains	Year of isolate	NT identity (%)	AA identity (%)	Genotypes
Hubei-China-KP772252	2014	99.30	99.88	I
Japan-AB178040	2004	98.14	99.59	I
Singapore-EU081257	2005	98.08	99.59	I
Singapore-EU081277	2005	98.36	99.59	I
Viet Nam-FJ410232	2007	97.69	99.44	I
Viet Nam-FJ461331	2008	97.68	99.38	I
Cambodia-GQ868619	2003	98.13	99.41	I
Thailand-HG316482	2010	97.51	99.44	I
Thailand-HM469966	2007	97.80	99.56	I
Malaysia-JN697057	2005	98.34	99.50	I
GuangDong-China-JQ048541	2014	98.51	99.65	I
Indonesia-KC762631	2008	97.88	99.50	I
Japan-LC016760	2014	97.95	99.44	I

**Table 3:** Non-synonymous amino acid mutations in the GZ02 strain compared to strains from other countries

AA	NT	B	Other countries
*E(936-2420)*			
581(E301)	1839	I	M
*NS3 (4521-6377)*			
1813 (NS3 338)	5535	R	G
1814 (NS3 339)	5538	D	G
1816 (NS3 341)	5544	P	S

**Fig. 2:**
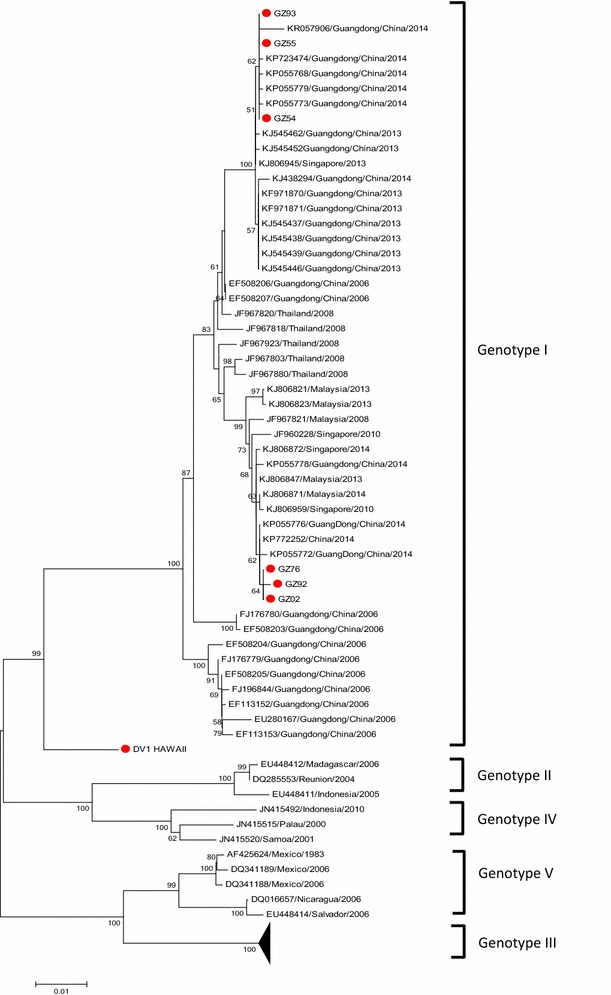
Phylogenetic tree of DENV-1 genotype I sequences based on the E gene. Phylogenetic relationships among GZ93.55.54.76.92.02 from different countries based on E gene were determined using the neighbour-joining method

**Fig. 3:**
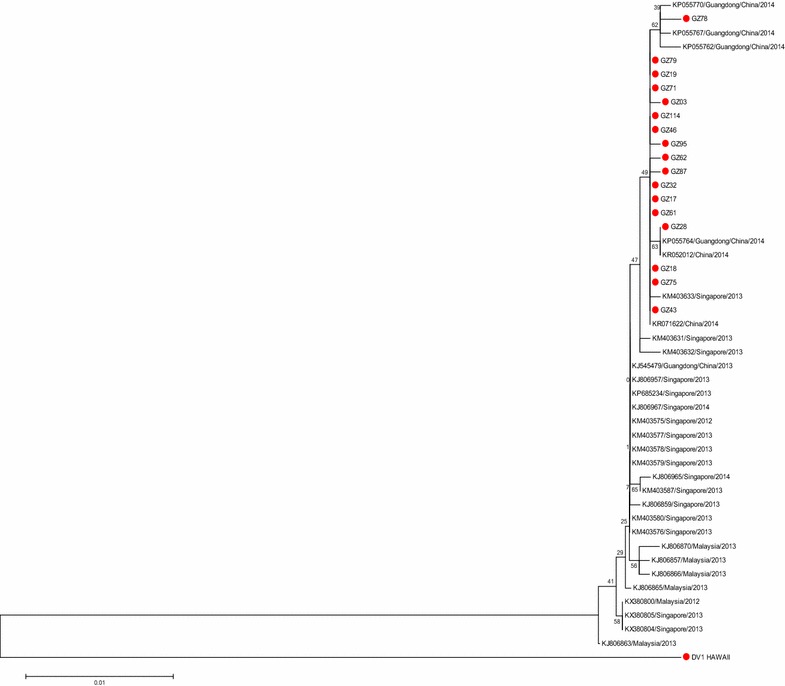
Phylogenetic tree of DENV-1 genotype III sequences based on the E gene. Phylogenetic relationships among GZ95.87.114.17.18.19.32.43.45.61.71.75.79.26.03.62.78 from different countries based on E gene were determined using the neighbour-joining method

In recent years, the sequences of dengue virus that have been uploaded mostly contain the E gene but fewer full-length genome. To clarify the origin of GZ02 further, the 23 DENV-1 strains (including GZ02) generated from the E gene were aligned with 1000 reference strains of various genotypes. GZ93 represents our new strains of genotype I were used to construct phylogenetic trees, After screening, a phylogenetic tree based on the E gene was constructed (Fig. 2). GZ95 was the representative strain of genotype III was used to Blast and phylogenetic analysis in the same way (Fig. 3). It showed that six strains clustered into DENV 1-I (genotype I), whereas the other 17 strains belonged to DENV 1-III (genotype III).

### Analysis of recombination events

Data from the RDP analyses suggested that there were only three recombination events between GZ02 and strains from Malaysia or Singapore (1226–1643, major parent: AY762084-Singapore-1993, minor parent: FJ196845-Guangdong-1991, *P* value: 3.657 × 10^−1^), (4614–4791, major parent: A75711-Singapore-1999, minor parent: EU081258-Singapore-2005, *P* value: RDP4.921 × 10^−3^, *P* value: siscan 2.891 × 10^−5^), (5347–5840, major parent: FJ196845-Guangdong-1991, minor parent: AY762084-Singapore-1993, *P* value: siscan 2.173 × 10^−1^). Some recombination events might have occurred in three DENV-1 isolates from Guangdong and Singapore. (FJ196847-Guangdong-1997, FJ196848-Guangdong-1999, and EF032589-Guangdong-2004) (Table 4), but no recombination event was found in any isolates collected after 2005. In addition, strains from Guangdong did not show any recombination events with any of the tested Malaysia strains (data not shown).

**Table 4:** Recombination analysis between Singapore and Guangdong strains

Strain	NT position	Major parent	Minor parent	Av.P-Val methods					
				RDP	GENECONV	MaxChi	Chimaera	SiScan	3Sep
FJ196847-Guangdong-1997	994–1701	EU081274	FJ196842	4.882 × 10^−9^	4.852 × 10^−9^	8.131 × 10^−9^	3.788 × 10^−9^	3.350 × 10^−17^	3.844 × 10^−40^
	2238–7197	EF025110	EU081234	3.415 × 10^−13^	7.161 × 10^−10^	7.79 × 10^−19^	3.318 × 10^−19^	1.389 × 10^−28^	9.458 × 10^−48^
	7617–8231	A75711	FJ196846	1.04 × 10^−2^	3.721 × 10^−4^	1.21 × 10^−4^	9.047 × 10^−5^	9.28 × 10^−9^	2.032 × 10^−4^
	8887–10675	FJ196844	FJ196846	6.537 × 10^−25^	7.002 × 10^−23^	3.198 × 10^−10^	1.354 × 10^−7^	7.451 × 10^−35^	4.79 × 10^−13^
FJ196848–Guangdong-1999	1187–1754	EU081274	FJ196842	4.882 × 10^−9^	4.582 × 10^−9^	80131 × 10^−9^	3.788 × 10^−9^	3.35 × 10^−17^	3.844 × 10^−4^
	1626–5978	EF025110	EU081234	3.415 × 10^−13^	7.161 × 10^−10^	7.79 × 10^−19^	3.318 × 10^−19^	1.369 × 10^−28^	9.458 × 10^−48^
	4604–4870	A75711	EU081258	4.921 × 10^−3^				2.891 × 10^−5^	
	7995–8851	KF971869	FJ196847	1.061 × 10^−2^	1.681 × 10^−4^	1.286 × 10^−2^	2.253 × 10^−3^	4.58 × 10^−13^	2.699 × 10^−2^
	8914–9201	GZ02	FJ196846	3.213 × 10^−4^	2.303 × 10^−6^			2.503 × 10^−8^	
EF032589-Guangdong-2004	1300–2514	EF025110	EU081234	3.415 × 10^−13^	7.161 × 10^−10^	7.79 × 10^−19^	3.318 × 10^−19^	1.369 × 10^−28^	9.458 × 10^−48^

### Comparison of the growth of DENV isolates in L02 cells

**Fig. 4:**
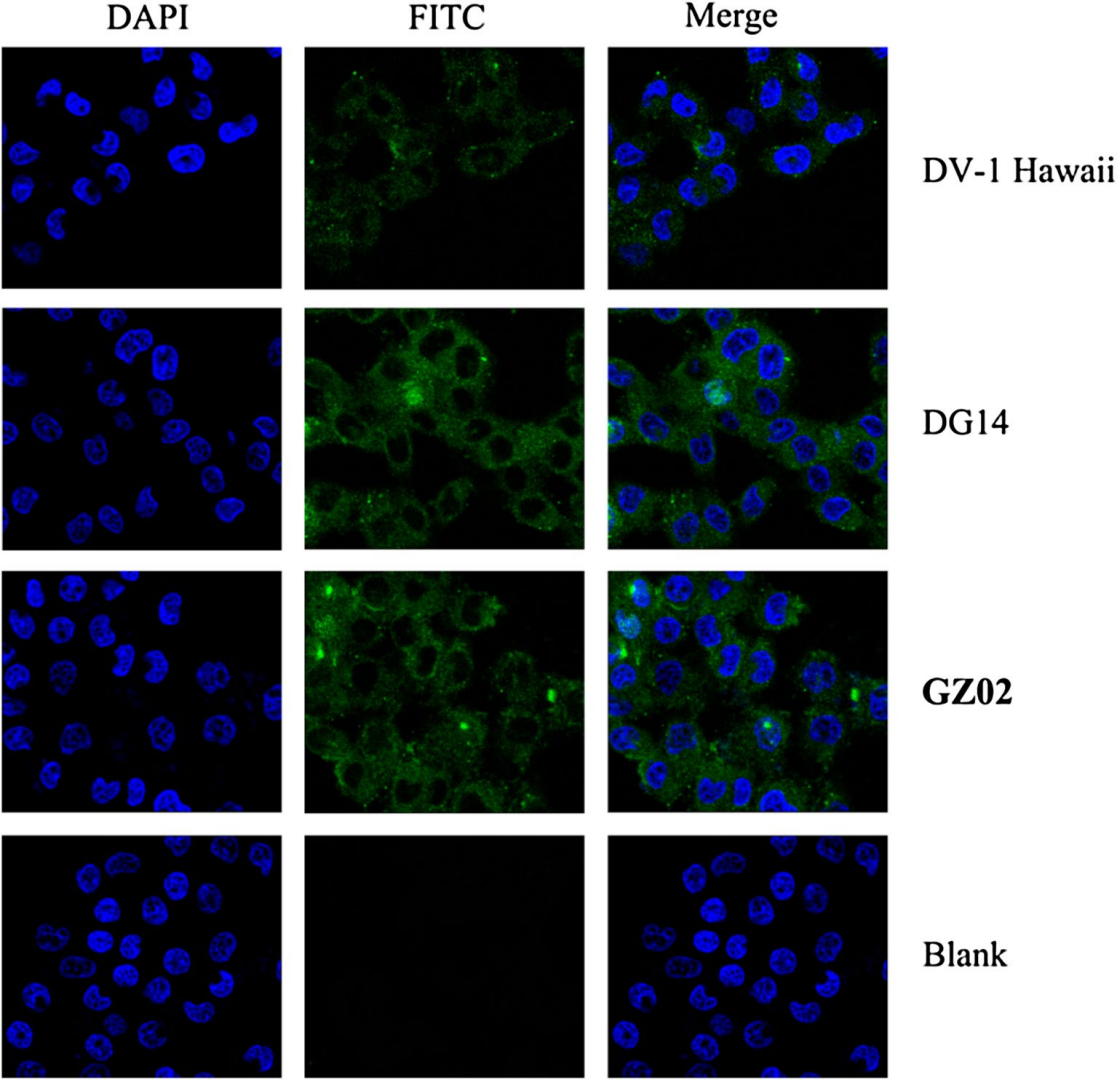
Infection of L02 cells by GZ02, DG14, and DV1 Hawaii visualized under a confocal microscope. *Green* indicates FITC-conjugated goat anti-mouse secondary antibodies binding to primary monoclonal mouse antibody 4G2. *Blue* indicates DAPI used for staining of nuclei. The images shown are representative of three independent experiments

**Fig. 5:**
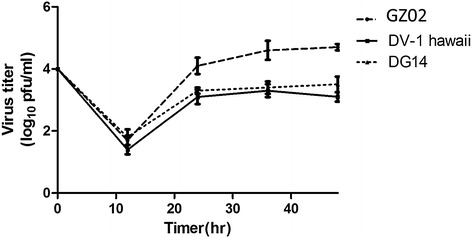
Growth curves of DENV strains GZ02, DG14, and DV-1 Hawaii in L02 cells. The culture supernatant of infected cells was collected at the following time points: 12, 24, 36 and 48 h. Analysis of variance (ANOVA) was used to test different growth rates between the GZ14 and the other two strains (*P* < 0.05)

Liver involvement in DENV infections has been well documented (Paes et al. 2009; Lin et al. 2000). Dengue virus can replicate and cause evident CPE in different liver cell lines (Sang et al. 2014). In our study, GZ02 was isolated from a 31-year-old female with no underlying diseases like viral hepatitis. Her liver function was abnormal. Alanine aminotransferase (ALT) levels reached 114 U L^−1^, which is approximately three times higher than the upper limit of normal; aspartate aminotransferase (AST) reached 152 U L^−1^, which was more than four times higher than the highest normal level; and serum albumin was slightly decreased at 32 g L^−1^. The DG14 strain, which was the strain most closely related to GZ02 from Guangdong, was not associated with liver damage (Peng et al. 2012). Replication kinetics of DG14, DENV-1 Hawaii (the standard strain), and GZ02 strains were compared in human normal liver cells L02. All of these strains were able to infect and replicate in L02 cells, as measured by indirect immunofluorescence (Fig. 4). To determine the infection efficiencies of the three strains, PRNT was performed. Statistical analysis indicated that DENV titres varied significantly between the isolates, and GZ02 showed significantly more growth than the other two strains at different time points (Fig. 5). GZ02 had a significant replicative advantage over DG14 and DV1 Hawaii at three of the four time points, excluding the time at 12 h post-infection (*P* < 0.05).

## Discussion

This study was conducted to molecular characterize and phylogenetically analyse strains from Guangzhou, Guangdong Province, in 2014. This was a large outbreak with 45,171 cases, including 37,354 cases in Guangzhou in 2014 (Health and Family Planning Commission of Guangdong Province 2014). Guangdong is one of the largest provinces in South China and DENV infections have been reported in Guangdong almost every year. DENV-1 was the dominant serotype circulating in Guangdong; however, all four serotype were in circulation. In recent years, Guangdong Province has had the highest incidence of dengue in China, and varying numbers of cases have been reported nearly every year since 1997 (Fan et al. 2014; Wang et al. 2015).

According to epidemiological data, strains from countries that had introduced DENV into Guangdong in the past few years were chosen for genetic analysis (Sang et al. 2015; Peng et al. 2012). Based on the percent nucleotide and amino acid identities to GZ02, one DENV-1 strain from each of these countries was chosen for phylogenetic analysis of the complete genome (Fig. 1). The phylogenetic tree based on full-length sequences indicated that GZ02 probably originated from Guangdong or a Southeast Asian country.

In recent years, most DENV sequences in GenBank were from the E gene only. To further determine the origin of GZ14 and the rest of the 22 new strains, a phylogenetic analysis based on the E gene was conducted (Figs. 2, 3). We found that GZ93.55.54 and GZ76.92.02 were in two different clades in genotype I. This analysis showed that GZ02, GZ76, and GZ92 were closely related to Malaysian and Singaporean strains from 2008 to 2013, indicating that these three strains likely originated from Malaysia or Singapore, rather than from Guangdong. Thailand was the probable source of this clade. The present study showed that this clade was the most common DENV-1 strain and that DENV1-I was the predominant genotype circulating in Malaysia (Ng et al. 2015). In the other clade, some strains in Guangdong in 2013 was widespread dissemination have a close link with GZ93, GZ55 and GZ54, and two strains in Guangdong in 2006 may be the probable source of this clade. The strains that invaded in 2013 persisted in Guangzhou to the following dengue season in 2014, which may be attributed to vertical transmission of DENV. Natural, vertical transmission of DENV has been shown to occur in *Aedes albopictus* mosquitoes in Guangzhou, suggesting that DENV-1 that cause the dengue outbreak in Guangzhou in 2013 may have been maintained locally until the outbreak in 2014 (Li et al. 2008). However, whether dengue fever is endemic in Guangdong remains controversial. Data from this study indicated that there may be persistence of the virus into the following epidemic season, suggesting two possibilities: (1) An endemic cycle of transmission has been established in Guangdong or (2) invading viruses have experienced severe bottlenecks and faded out at the end of each transmission season, and as a result, DENV are reintroduced annually to Guangdong from other countries. Importantly, however, the virus only persisted for two years, and whether in situ evolution occurs, remains to be seen. The remaining 17 new strains belonged to genotype III and were closely related to outbreak strains from Malaysia and Singapore in 2013 (Fig. 3). It was shown that Malaysia and Singapore are hyperendemic of dengue virus circulating in the country. Figure 3 indicated that the outbreak in Guangdong in 2014 may probably import from Malaysia and Singapore. Furthermore, some 2014 strains were co-circulating in Malaysia, Singapore, and Guangdong, indicating that frequent exchanges of genetically identical DENV were shared among the three countries.

In recent decades, among the cases of DENV infection in Guangdong, most of the imported cases have originated from Southeast Asian countries (Sun and Meng 2013). Most of the viruses in this study originated from Southeast Asia countries that have close ties with Guangdong. Dengue is a disease endemic in most Southeast Asian countries, and these countries bear nearly 75% of the current global burden of this disease (Roehrig et al. 2008). Guangdong Province has an appropriate environment for DENV transmission and this city has strong political and economic contacts with these countries (Chen 2011; Hu et al. 2011). Although multiple genotypes of DENV-1 have been detected, no monophyletic clade was formulated and circulated in Guangdong in 2014. Because of the lack of significant evidence that DENV is endemic in Guangzhou, we speculate that the 23 DENV strains likely originated from the Southeast Asian region. Based on this study, we still believe that DENV is an imported disease in Guangdong.

In many RNA viruses, recombination and reassortment are known to be highly associated with evolution. Although some recombination events might have occurred between DENV-1 isolates, recombinations between strains from Guangdong and Singapore or Malaysia seem to have been very limited. Only 52 out of 440 strains (12%) showed a high probability of having undergone a recombination event. Recombination between strains from Singapore and Guangdong was seen in only 3 of the 440 strains included in this study, which may be attributed to the large unprecedented outbreak of DENV-1 in Singapore in 2005 (Koh et al. 2008). Furthermore, no recombination events occurred between strains from Guangdong and Singapore or Malaysia after 2005, which may support the idea that DENV are reintroduced to Guangdong from Southeast Asia, but have not been established locally. Survival and localized transmission of an introduced DENV strain in a particular region is dependent on a combination of factors, such as density of mosquito population, biting frequency, and the period of cross-immunity. We speculated that strains from Singapore or Malaysia cannot overwinter and circulate in Guangdong in subsequent years. This lack of year-round transmission locally might reduce the possibility of recombination events, despite frequent contact between Guangdong and Singapore or Malaysia.

DENV infects a variety of cell types, leading to a spectrum of clinical and pathological changes. It mainly affects muscles and the circulatory system. However, common clinical and experimental observations indicate that a majority of dengue cases are associated with abnormal liver enzyme levels (Lin et al. 2000). Liver damage in patients with dengue may be caused directly by virus infection of liver cells or by dengue virus–mediated host immune responses. Indirect immunofluorescence assay showed that L02 cells can be infected by DENV strains from different regions, including DG14, DV1 Hawaii (The standard strain), and GZ02. GZ02 showed the greatest virulence and highest replication in L02 among the DV1 strains. Therefore, this strain may induce severe liver damage with the elevation of ALT and AST, as well as a reduction in triglycerides. However, the molecular mechanism by which GZ02 has a significant replicative advantage over other DENV-1 strains remains to be elucidated. Four non-synonymous amino acid substitutions, E301, NS3 338, NS3 339, and NS3 341, may have affected viral function and increased the ability of the virus to infect and replicate in liver cells.

## Conclusions

Since no effective medicine or preventive vaccine is available for dengue, prevention and control measures are critical. Determining the origin of the 2014 strains from Guangzhou might provide information that could lead to more effective control measures. Our results indicate that DENV-1 transmission cycle in Southeast Asian countries, especially Singapore and Malaysia, may have played a significant role in the dengue outbreak that occurred in Guangdong in 2014. However, the origin and the impact of these new strains in Guangdong require further consideration.

## Methods

### Virus isolation

GZ02 was isolated from the serum of a clinical case of dengue fever in Guangzhou, China, in 2014. The serum was inoculated into BHK-21 cells for virus isolation, and was cultured in RPMI-1640 medium supplemented with 2% fetal bovine serum at 37 °C in 5% CO_2_. The cells were observed for 6 days for cytopathic effect (CPE), and virus was harvested from culture supernatant.

### Genome sequencing and analysis

A viral RNA Mini Kit (Qiagen, Germany) was used to extract viral RNA. Twelve pairs of primers were designed to amplify the complete genome of GZ02, and four pairs of primers were designed to amplify the E gene of the 23 new strains (Table 5). The amplification process was carried out as follows: an initial denaturation (95 °C, 5 min), 35 cycles of denaturation (94 °C, 30 s), annealing (56 °C, 1 min), extension (72 °C, 1 min), and final extension (72 °C, 10 min). Agarose gel electrophoresis (2%) was used to analyse the polymerase chain reaction (PCR) products, which were then sequenced by a commercial facility (IGE Biotechnology Ltd, Guangzhou, China).

**Table 5:** Nucleotide sequence of primers used in the PCR assay

Name	Forward primer sequence (5′ → 3′)	Position	Name	Reverse primer sequence (5′ → 3′)	Position
1F	GTTAGTCTACGTGGACCGAC	6–25	1R	CCAACACCACATCTACCCAA	992–1011
2F	TAGCACATGCCATAGGAACATC	856–887	2R	CCTTCGTATTTAACCTGCACTAG	1896–1918
3F	TCACAAGAAGGAGCAATGCACA	1698–1719	3R	AAGAAGAACTTCTCTGGATGTTA	3763–3785
4F	TGGAGCCAACGCTTCCGACA	3651–3675	4R	TCCCAGGAGACCTCAGCCGC	4296–4315
5F	ATATCTGGAAGCTCAGCCGA	4260–4279	5R	CCCTGGTTCAACAGCAATCA	4810–4829
6F	CTGTACTCATGTATCAAGGGAAGA	4687–4710	6R	CTTGGATAACTGCGTTGCTCTG	5502–5523
7F	AGGGAATGCCAATAAGGTAC	5230–5249	7R	GTCCTGCTAAGATGACACGC	5840–5859
8F	CAGAGCAACGCAGTTATCCA	5502–5521	8R	CAATTTAGCGGTTCCTCTCG	7741–7760
9F	GCAAGTCAGAATTCAACACC	7636–7655	9R	CATATGATCCATGATAGGCC	8480–8499
10F	CGATTCACAATGGCTCACAGGA	8295–8316	10R	ATGGCACCACTATTTCCCTCCC	9732–9753
11F	CTAACCTACCAAAATAAAGT	9383–9301	11R	TTAGTCTTCTCACTTGGTTT	10,172–10,191
12F	CCATATTTAGGGAAAAGGGA	10,080–10,099	12R	CGCCTGGAATGATGCTGTAG	10,672–10,691
E 1F	TAGCACATGCCATAGGAA	856–873	E 1R	CTGGGTCTCAGCCACTTC	1866–1883
E 2F	ATGCAAAGAAGCAGGAAG	1666–1683	E 2R	AATTTGTATTGCTCTGTCCA	2502–2521
E 3F	CATAGGAACATCCATCACC	866–884	E 3R	TATTGCTCTGTCCAAGTGTG	2496–2515
E 4F	GAACATCCATCACCCAGAAA	871–890	E 4R	GCTCTGTCCAAGTGTGAACTT	2491–2511

The sequence was spliced using SEQMAN from the LaserGene package (DNASTAR Inc., Madison, WI). The nucleotide and amino acid sequences were aligned using the Clustal X multiple sequence alignment program. DENV reference sequences were downloaded from the GenBank database.

### Phylogenetic analysis

A phylogenetic tree based on full-length sequences of DENV was constructed from a dataset including isolate GZ02 and 1000 reference sequences downloaded after the Blast analysis. One representative strain from each country that was considered a likely source for DENV introduction into Guangdong was chosen to construct the final phylogenetic tree.

A total of 1000 reference strains of various genotypes and 23 new strains (GZ02 included) were used in a phylogenetic analysis of the E gene, in combination with the top 250 best-match sequences of each new strain. After screening, a phylogenetic tree based on the E gene was constructed using 83 strains from different countries. The phylogenetic analysis based on full-length and E gene sequences was carried out using the neighbour-joining method in MEGA version 6.06 (http://www.megasoftware.net/). Numbers at nodes represent significant bootstrap percentages (>70%) and the bar indicates substitutions per site. A bootstrap test with 1000 replications was used to show the reliability of the analysis.

### Indirect immunofluorescence and plaque reduction neutralization testing for the detection of DENV

Titers of DENV-1 strains GZ02, DV1 Hawaii (the standard strain), and DG14 were determined by plaque assay in BHK-21 cells as previously described (Othman et al. 2010). Viruses were prepared at 1 × 10^4^ PFU ml^−1^ and divided into vials containing 0.5 ml aliquots each to avoid thawing and refreezing of viruses. After infection with GZ02, DV1 Hawaii, and DG14, each at a multiplicity of infection of 1, human normal liver cell L02 were collected at 12, 24, 36, and 48 h and were fixed with 4% paraformaldehyde for 15 min. Then, L02 cells were made permeable by incubating with Fixation/Permeabilization reagent (Becton, Dickinson and Company, USA) for 20 min at 4 °C. Monoclonal antibody (mAb) 4G2 and rabbit anti-mouse antibody conjugated to fluorescein isothiocyanate (FITC) (Proteintech Group, USA) were used as the primary and secondary antibody, respectively, to detect dengue virus in L02 cells. After washing by phosphate buffered saline with Tween-2 (PBST), the cell nuclei were stained with 4′,6-diamidino-2-phenylindole dilactate (DAPI) (Beyotime, China) for 5 min and then examined under a confocal microscope (Olympus, Japan).

### Recombination analysis

The RDP, GENECONV, MAXCHI, CHIMAERA, SISCAN, and 3SEQ methods, implemented in the program RDP4, were used to detect discrete recombination events. Recombination breakpoint positions and recombinant/parental designations were inspected and adjusted as needed using the phylogenetic and recombination signal analysis features in RDP4.
